# The relationship between exposure to long-term training, neuromuscular function and muscular structure in adolescents with cerebral palsy and typically-developed peers: a cross-sectional follow-up analysis

**DOI:** 10.1186/s12891-026-09829-3

**Published:** 2026-05-01

**Authors:** Alice Minghetti, Ralf Roth, Sereina Büttiker, Eric Lichtenstein, Paul Ritsche, Martin Keller

**Affiliations:** Department of Sport, Exercise and Health, University of Basel, Basel, Switzerland

**Keywords:** neuromuscular, exercise, strength, function, exercise training, youth

## Abstract

**Background:**

This cross-sectional study examined whether exposure to long-term resistance and endurance training can counteract muscular weakness on a functional, neurological and structural level in adolescents with cerebral palsy (CP) compared to typically-developed peers (TD) depending on training status.

**Methods:**

Five trained (4 males; mean age: 19.8) and four untrained adolescents with CP (3 males; 20.2) were compared to nine age- and sex-matched TD trained (7 males; 19.8) and nine untrained peers (7 males; 20.3). Isometric and isokinetic measurements assessed strength in knee flexion and extension, voluntary activation (VA) was assessed using the twitch interpolation technique and ultrasound imaging of the quadriceps was performed to assess anatomical cross-sectional area (ACSA) and architecture.

**Results:**

Linear regression models revealed that CP trained had lower absolute isometric strength (dominant: -18% [-48; 11]; non-dominant: -35% [-58; -11]) than TD untrained while CP untrained showed between 29% and 33% lower strength than TD untrained. VA in CP trained (dominant: -13% [-23; -3]; non-dominant: -10% [-30; 11]) and CP untrained (dominant: -14% [-23; -4]; non-dominant: -8% [-29; 13]) showed similar deficits compared to TD untrained. CP trained showed higher ACSA than TD untrained in the dominant leg of the vastus lateralis muscle (+ 16% [-7; 38]), while the non-dominant side showed lower values (-18% [-45; 9]).

**Conclusion:**

Exposure to long-term resistance and endurance training is associated with a reduced gap in muscle strength and muscle volume in the dominant leg of adolescents with CP while neural drive does not seem to be affected through training exposure. It is discussed that training load might have been too low in the non-dominant leg of CP trained to induce relevant neuromuscular adaptations.

**Trial registration:**

ClinicalTrials.gov Identifier NCT05859360, date of registration May 16, 2023.

**Supplementary Information:**

The online version contains supplementary material available at 10.1186/s12891-026-09829-3.

## Background

 Cerebral palsy (CP) is a group of permanent disorders, attributed to non-progressive disturbances occurring in the developing fetal or infant brain [1]. CP affects the development of movement and posture, causing activity limitations and is oftentimes accompanied by secondary musculoskeletal problems [1]. The motor disorders associated with CP during childhood are a major source of concern, as they can lead to increased spasticity, joint contractures, joint pain and deteriorating muscle tone as well as increasing fatigue and inefficiency or inability of gait in adulthood [2].

One of the main causes of many of these disturbances is the inability to produce force, directly impacting everyday function. There have been vast collections of evidence supporting the finding that children with CP are weaker than their typically-developed (TD) peers, independently of spasticity or severity of the disorder [3, 4]. Reduced strength has been attributed to factors such as reductions in muscle size [5–7], an increased activation of antagonistic muscles [8], and an incomplete activation of agonists [6, 9, 10]. Neural factors contributing to clinical weakness in CP include reduced motor drive, reinforcement of abnormal neural circuits, altered recruitment patterns, impaired reciprocal inhibition and altered settings of muscle spindles [6, 11]. For example, it has been described that individuals with CP show large deficits in the level of voluntary activation when compared to healthy controls [9, 12]. A sufficient level of voluntary activation is likely to be relevant for resistance training, as those individuals with CP with substantial deficits in voluntary muscle activation may be unable to generate sufficient force to elicit muscle hypertrophy through voluntary contractions. Thus, there is a strong need to investigate whether long-term training can effectively enhance muscle mass and voluntary activation in individuals with CP.

Besides impaired neuromuscular activation, reduced muscle size is a major contributor for strength impairments [5–7]. However, the effects of the disorder on muscle geometry are only scarcely investigated. There is inconsistent evidence showing altered indicators of muscle morphology and muscle architecture – both subunits of the muscle geometry – in patients with CP in dependence of the examined muscle and the form of CP. One of the only consistencies in studies examining patients with spasticity lies in increased fiber size variability, increased numbers of rounded fibers and “moth-eaten” fibers [13]. Examinations in children with CP have been able to show shorter fascicles and smaller cross-sectional area of muscle fibers in the quadriceps muscles, indicating alterations which occur secondary to the neural lesions and which are generally observed with disuse and aging [14, 15]. A further determining criteria for force production is muscle anatomical cross-sectional area (ACSA). Children with CP show lower ACSA than TD peers, especially in muscles essential for locomotion [6, 16] which can also, in part, explain their lower force and strength production [14, 15]. Furthermore, smaller pennation angles (PA) as well as higher intramuscular fat tissue has been observed in children with CP compared to TD peers, which could contribute to a reduced contractile ability of muscle tissue [6, 16, 17]. It remains unclear whether these abnormalities are due to their disorder or are a secondary consequence to their reduced neuromuscular control and corresponding inactivity levels.

CP is the most common form of childhood disability, and measures are needed to combat the neuromuscular impairments and the resulting secondary musculoskeletal challenges, including inactivity, muscle weakness and altered muscle geometry [2]. Training recommendations for patients with CP have been formulated, proposing a combination of high-intensity resistance and endurance training [18]. However, there are a variety of barriers to effective participation, ranging from emotional and environmental to economic factors [19]. Nevertheless, the positive effects of resistance training on muscle function has been successfully demonstrated in feasibility studies [20] but also randomized controlled trials [21, 22]. However, gains in muscle strength obtained from an intervention trial with just a few weeks of training may not affect general mobility [22]. In addition, detraining effects have been described early after the end of a training intervention [22]. Thus, there is currently no evidence for any long-term effects of guideline-based training on neuromuscular activation, strength and muscle geometry in CP populations, especially when compared to TD peers.

This cross-sectional follow-up study compared maximum strength, voluntary activation as well as muscle geometry of trained and untrained adolescents with CP to trained and untrained TD peers. The aim of the study was to examine whether exposure to long-term, high-intensity resistance and endurance training can counteract weakness on a functional, neurological and structural level in adolescents with CP. We hypothesized that long-term participation in high-intensity training including strength and endurance positively affects muscle mass, muscle function but also neuromuscular activation in individuals with CP. We expected higher performance when comparing trained to untrained individuals with CP but impaired performance in trained individuals with CP when compared to trained but also untrained healthy peers.

## Methods

### Study design and populations

This cross-sectional study was approved by the local ethics committee (Ethikkommission Nordwest- und Zentralschweiz: 2023 − 00578) and was in accordance with the Declaration on Helsinki and Good Clinical Practice. The current analysis compares four age- and sex-matched groups: (a) Trained with cerebral palsy (CP trained, *n* = 5, all unilateral, all GMFCS level I); (b) Untrained with cerebral palsy (CP untrained, *n* = 3 unilateral and *n* = 1 ataxia, *n* = 3 GMFCS level 1 and *n* = 1 GMFCS level II) ; (c) Typically-developing trained (TD trained, *n* = 9); and (d) Typically-developing untrained (TD untrained, *n* = 9).

All individuals with CP were participants of a feasibility study and had undergone a rigorous screening process described elsewhere [20, 23]. Eight were classified level I of the Gross Motor Functional Classification System (GMFCS) while one was classified as level II. Adolescents who continued to attend the high-intensity training program (between once and twice a week with each session lasting 60 min) over the past 2.5 years were assigned to the CP trained group. Detailed information about the training can be found in the feasibility study [20]. In short, each training session started with a 10 min warm-up followed by a 30 min circuit including strength exercises (focusing on pulling, pushing, legs). Each training session ended with a 12–15 min high-intensity workout with the aim of maximum cardio-respiratory exhaustion. Some of the participants of the feasibility study could not continue the training program due to time and transportation issues (e.g. distance to training location) and therefore returned to standard care. Those individuals were allocated to the CP untrained group. TD peers had to be of same age and sex as an individual of the CP trained or untrained group and were recruited through personal contacts. Exclusion criteria were the diagnosis of congenital heart defects, pulmonary diseases, neuromuscular disorders or any acute diseases. Training status was assessed in all participants using the International Physical Activity Questionnaire (IPAQ) [24]. Individuals who did not participate in any sports or physical activity programs beside school sports (i.e. more than two hours of structured exercise exposure per week) were allocated to the TD untrained group. Adolescents who were active in any additional form of sports program or activity were assigned to the typically-developed trained group. Participants in the TD-trained group were recruited primarily on the basis of regular physical activity, with less emphasis on a specific training modality. Among participants in the TD-trained group, the main sports were CrossFit (*n* = 1), soccer (*n* = 3), swimming (*n* = 1), volleyball (*n* = 2), and athletics (*n* = 2). Weekly MET-minutes ranged from 3,458 to 10,476, and all participants in this group were therefore classified as highly active. Cohort characteristics can be found in Table 1. Measurement staff were blinded to subgroup allocation of the adolescents with CP as well as typically-developed peers.

**Table 1:** Summary of study population. Data shown as mean with standard deviation (SD)

	CP Trained (*n* = 5)	CP Untrained (*n* = 4)	TD Trained (*n* = 9)	TD Untrained (*n* = 9)
Characteristics				
Age [years]	19.8 (3.0)	19.9 (5.7)	19.8 (4.2)	20.3 (4.4)
Sex [m/f]	4/1	3/1	7/2	7/2
Anthropometry				
Height [cm]	174 (9.6)	166 (7.1)	177 (7.3)	175 (8.9)
Body mass [kg]	80.9 (32.1)	79.7 (19.4)	71.2 (14.8)	76.4 (16.0)
Skeletal muscle mass [kg]	34.3 (8.0)	28.8 (5.5)	34.4 (7.3)	31.2 (6.9)
Relative fat mass [%]	21.5 (13.7)	33.2 (12.1)	14.3 (7.7)	25.6 (11.3)
BMI [kg/m^2^]	26.4 (9.6)	28.6 (5.6)	22.6 (3.7)	25.4 (7.5)
Physical activity				
Moderate-to-vigorous exercise per week [min]	194 (110.5)	59 (54.9)	503 (531.4)	107 (123.6)
Form of Cerebral Palsy				
Unilateral [n]	5	3	Not applicable	
Ataxia [n]	0	1	Not applicable	
Gross Motor Function Classification System (GMFCS)				
Level I [n]	5	3	Not applicable	
Level II [n]	0	1	Not applicable	

### Procedures

Maximum strength tests were performed for both legs using the Isomed^®^ 2000 dynamometer (D&R Ferstl GmbH, Hemau, Germany). Maximum strength was assessed for knee extensor and knee flexor muscles after a brief familiarization of the participants to the testing procedure. Participants were strapped to the device with a hip angle of 75° and a knee angle of 60° with the adapter placed at 2/3 of the distance between the knee joint and the lateral malleolus [25]. For isokinetic assessment of knee flexion and extension at a speed of 60° per second, participants were instructed to push and pull the lever arm as hard and as fast as possible over the entire range of motion (0–90°, 0° knee fully extended) for five repetitions. Two sets were performed with one minute of rest between sets. For isometric knee extension, participants were instructed to push as fast and as hard as possible against the immovable adapter for 3–5 s. The test was conducted three times with one-minute breaks between trials. Peak torque from all three knee extension trials (at 60° knee flexion) were extracted and normalized to participants’ body weight. Also, peak rate of force development (RFD) over 200 ms time frames were calculated from the force-time data and used to measure explosive strength.

Voluntary activation (VA) was assessed and quantified by the interpolated twitch technique by means of electric muscle stimulation. The stimulation procedure was applied to both legs, with the dominant leg being measured first. All procedures were based on a recent Delphi survey expert consensus [26]. Electrical stimulations were delivered transcutaneously using neurostimulation surface electrodes (Axelgaard Manufacturing Co., Ltd., Denmark) connected to a constant current stimulator (DS7AH, Digitimer, Hertfordshire, UK). Stimulation electrodes were placed on the proximal and distal part of the quadriceps muscle. Participants were seated in a dynamometer (Knee Dynamometer, S2P science to practice, Ljubljana, Slovenia) with their knee and hips fixated at a 90° joint angle and the adapter placed at 2/3 of the distance between knee and ankle joint. To determine the individual stimulation intensity, electrical stimulation (1 ms pulse width) was gradually increased in steps of 50 mA. The optimal intensity was considered to be reached when no further increase in peak twitch force was observed. The stimulation intensity was then increased by another 20% to ensure supramaximal stimulations during data assessment. Participants were familiarized with the isometric knee extensions by performing 8–10 submaximal contractions (20–80% of their self-estimated MVC). After this warm-up phase, participants performed maximum isometric voluntary contractions. Electrical doublet stimulations (1 ms pulse width, 10 ms interstimulus interval) were applied during and approximately 4 s after the contractions to evoke superimposed and resting twitches, respectively. Five VA measurements were conducted and the three best values were used for analysis. VA was quantified using the following formula [26]:

$$\begin{array}{ll}\:voluntary\:activation\:\left(\%\right)=\:1\lfloor-\frac{superimposed\:twitch}{resting\:potentiated\:twitch}\rfloor\times\:100\end{array}$$

Due to one participant not being able to perform the resting twitch in one leg due to discomfort, central activation ratio was also calculated with the following formula [26]:

$$\begin{array}{ll}\:central\:activation\:ratio\:\left(\%\right)=\\\:\lfloor\frac{MVC\:force}{MVC\:force+superimposed\:twitch}\rfloor\end{array}$$

Anatomical cross-sectional area (ACSA), muscle thickness (MT), fascicle length (FL) and pennation angle (PA) of the m. vastus lateralis and ACSA of the m. rectus femoris were measured in both legs using B-Mode ultrasonography (ACUSON Juniper, SIEMENS Healthineers, Erlangen, Germany) with a 5.6 cm, linear-array probe (6.2–13.3 MHz, 12L3, Acuson 12L3). Participants lay in a supine position with their hips and knees fully extended. Three images of the m. vastus lateralis were recorded with the transducer orientated perpendicular to the skin and aligned with the fascicle plane. MT, FL and PA were assessed at 50% of the femur length. MT is defined as the distance between the superficial and deep aponeurosis of the muscle. PA is defined as the angle of insertion of the fascicle into the deep aponeurosis. The images were analyzed using the DL Track US python package (v0.2.1) and all automatic segmentations were visually inspected. Here, FL and PA are calculated as the respective median value of all detected fascicles subsequent to filtering of overlapping fascicles [27]. MT is calculated at 50% of aponeurosis width. Final muscle architectural values of both analyzed images were averaged for further analysis. Two panoramic ultrasonography images of the m. rectus femoris and m. vastus lateralis were recorded at 50% of femur length. The transducer was orientated perpendicular to the skin in the transversal plane and a guide was used to ensure a consistent imaging plane. Images were analyzed using the DeepACSA python package (v0.3.1) and all automatic segmentations were inspected [28]. The ACSA estimates of both analyzed images were averaged for further analysis. In case of erroneous segmentation, we used the ACSAuto (v1.4.0) FIJI plug-in for semi-automatic segmentation [29, 30]. All images were acquired by experienced operators with more than three years of experience.

### Statistical analysis

Data of all groups is shown as mean with standard deviations (SD). Individuals from the TD untrained group were regarded as the reference group and data for all participants in the other three comparator groups (CP trained, CP untrained, TD trained) were compared to their respective match in the reference group (TD untrained) by calculating the difference in values to the match. This was done so that non-matched participants were not compared to each other within the model. Linear regression models were calculated on the matched-differences. Between-group differences are presented as percentages (%) with 95% confidence intervals (95% CI) compared to the TD untrained reference group. In addition, general linear models were calculated to assess how much of the variance in the outcomes can be explained by training status (trained vs. untrained) and health status (TD vs. CP).

## Results

### Influence of training status and health status on performance

The general linear models revealed that 38.0% of the variance in isometric strength (normalized to body weight) is explained by health status and to 15.4% by training status. With respect to ACSA, 16.0% of the variance is explained by training status and 2.9% by health status. The analysis for voluntary activation revealed the 31.5% of the variance can be explained by health status with no influence of training status (0.0%).

### Maximum strength

**Fig. 1:**
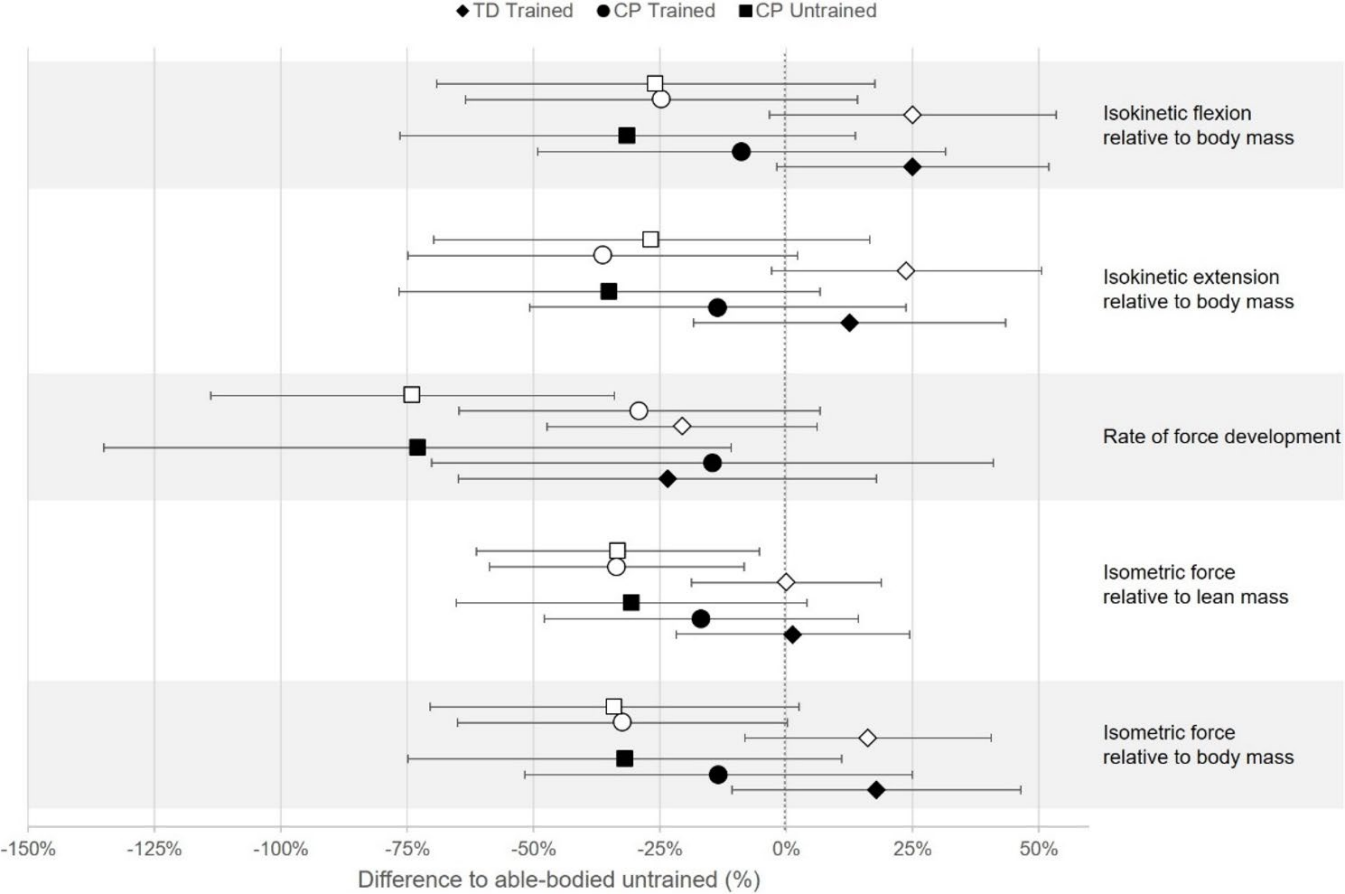
Between-group differences for isometric and isokinetic knee extension from linear regression model. Parameters shown as differences in percent to typically-developed untrained group. Dominant leg = black; Non-dominant leg = white; TD = typically-developed; CP = cerebral palsy

CP trained showed lower absolute isometric strength (dominant: -18% [-48; 11]; non-dominant: -35% [-58; -11]) as well as relative strength values (dominant: -13% [-52; 25]; non-dominant: -32% [-65; 0]) compared to untrained TD, whereby the differences were more pronounced in the non-dominant compared to their dominant leg (Fig. 1). Worst strength performance was found in the CP untrained group, where their absolute values were between 29% and 33% less than TD untrained peers with the difference increasing when set in relation to body mass (dominant: -32% [-75; 11]; non-dominant: -34% [-71; 3]). Differences between dominant and non-dominant leg were not evident in the CP untrained group. TD untrained showed highest RFD with CP trained reporting − 15% [-70; 41] in their dominant leg and − 29% [-65; 7] in their non-dominant leg. Differences between dominant and non-dominant leg were not apparent in either the TD trained (-24% [-65; 18] vs. -21% [-47; 6]) nor in the CP untrained group (-73% [-135; -11] vs. -74% [-114; -34]).

Isokinetic measurement of knee extension showed highest performance in TD trained (dominant: +13% [-12; 43]; non-dominant: +24% [-7; 50]). CP untrained showed worst performance in both legs (dominant: -35% [-75; 4]; non-dominant: -27% [-70; 16]) while CP trained showed worst performance in their non-dominant leg (-36% [-75; 2]) while the difference diminished in their dominant one (-14% [-51; 24]). CP trained showed diminished knee flexion strength than TD untrained, whereby differences were larger in the non-dominant (-25% [-64; 14;]) than dominant leg (-9% [-49; 32]). Strength data is shown in Table 2; Fig. 1 while data from linear regressions can be found in *Supplementary Material*.

**Table 2:** Data from isometric knee extension and isokinetic knee extension and flexion. Data for the dominant (non-affected) and non-dominant (affected) leg shown as mean with standard deviation (SD)

	CP Trained (*n* = 5)		CP Untrained (*n* = 4)		TD Trained (*n* = 9)		TD Untrained (*n* = 9)	
Isometric extension 60°	Dominant	Non-dominant	Dominant	Non-dominant	Dominant	Non-dominant	Dominant	Non-dominant
Max. strength [Nm]	209 (67)	159 (53)	138 (41)	122 (32)	246 (76)	225 (61)	229 (72)	216 (66)
Strength relative body mass [Nm/kg]	2.7 (0.8)	2.0 (0.4)	1.8 (0.5)	1.6 (0.5)	3.4 (0.5)	3.2 (0.4)	2.9 (0.9)	2.7 (0.8)
Strength relative lean mass [Nm/kg lean mass]	3.4 (0.6)	2.6 (0.4)	2.6 (0.4)	2.3 (0.4)	4.0 (0.7)	3.7 (0.5)	4.0 (1.0)	3.7 (0.8)
RFD [Nm/ms]	5.1 (1.6)	3.4 (1.7)	1.9 (1.0)	2.3 (1.1)	4.7 (0.7)	4.5 (0.9)	6.1 (3.0)	5.7 (1.9)
Isokinetic extension / flexion	Dominant	Non-dominant	Dominant	Non-dominant	Dominant	Non-dominant	Dominant	Non-dominant
Extension relative body mass [Nm/kg]	2.2 (0.4)	1.6 (0.4)	1.3 (0.6)	1.2 (0.5)	2.7 (0.6)	2.6 (0.5)	2.4 (0.7)	2.1 (0.8)
Extension relative lean mass [Nm/kg lean mass]	2.8 (0.3)	2.1 (0.3)	2.0 (0.8)	1.8 (0.7)	3.2 (0.6)	3.0 (0.6)	3.2 (0.9)	2.9 (1.0)
Flexion relative body mass [Nm/kg]	1.2 (0.3)	1.1 (0.4)	0.7 (0.3)	0.7 (0.4)	1.5 (0.4)	1.5 (0.2)	1.2 (0.4)	1.2 (0.4)
Flexion relative lean mass [Nm/kg lean mass]	1.6 (0.3)	1.3 (0.3)	1.1 (0.4)	1.1 (0.6)	1.7 (0.3)	1.8 (0.2)	1.7 (0.6)	1.7 (0.4)
Hamstrings: Quadriceps ratio [%]	56 (8)	65 (21)	56 (11)	57 (11)	55 (8)	60 (12)	53 (8)	61 (19)

### Voluntary activation

**Fig. 2:**
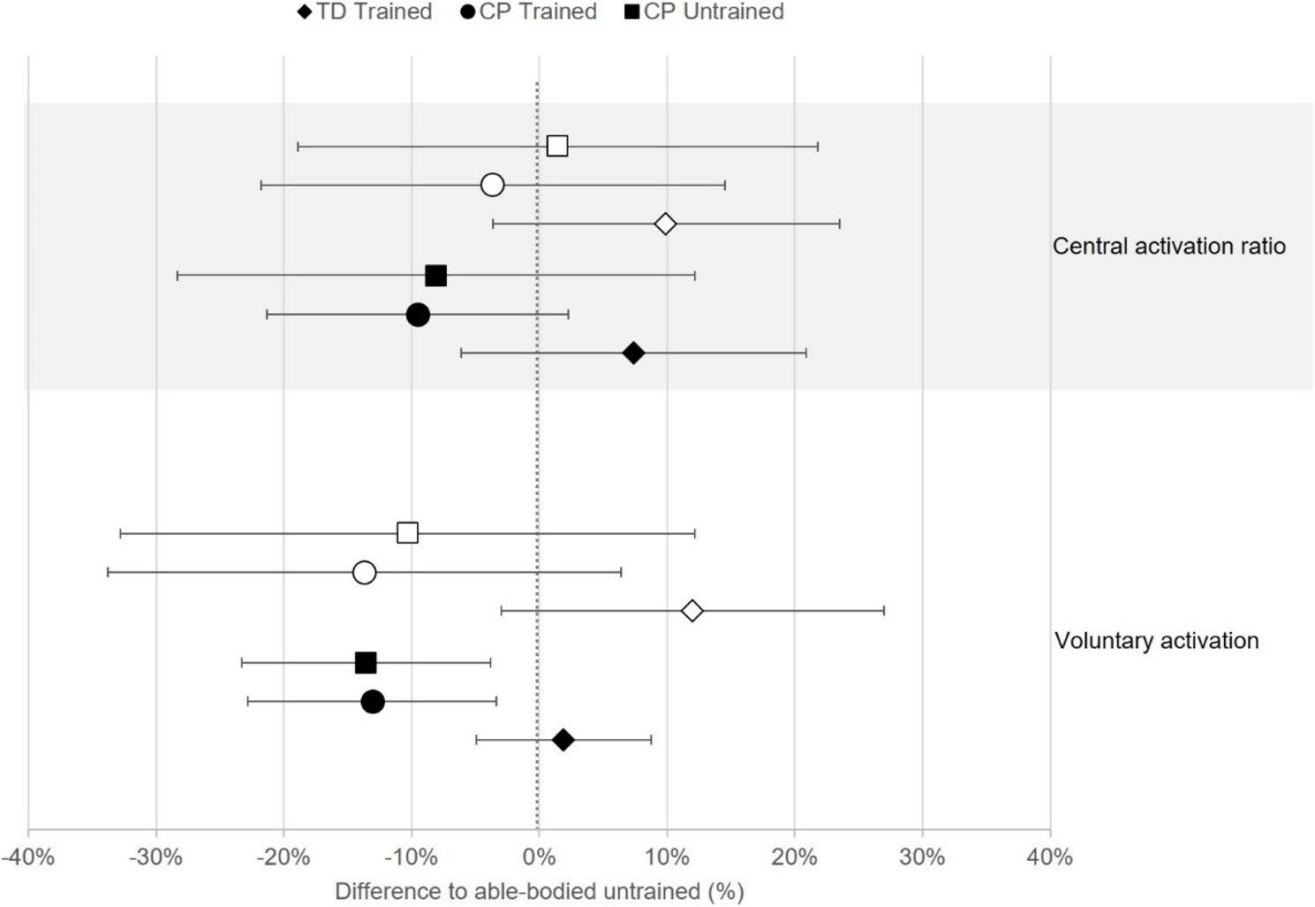
Between-group differences voluntary activation and central activation ratio from linear regression model. Parameters shown as differences in percent to typically-developed untrained group. Dominant leg = black; Non-dominant leg = white; TD = typically-developed; CP = cerebral palsy

Voluntary activation levels were highest in the TD trained group in both legs (dominant: +2% [-5; 9]; non-dominant: +7% [-7; 22]) compared to TD untrained. CP trained showed worse values (dominant: -13% [-23; -3]; non-dominant: -10% [-30; 11]), with similar deficits as the CP untrained (dominant: -14% [-23; -4]; non-dominant: -8% [-29; 13]). Central activation ratio was found to be similar on the dominant side (+ 1% [-26; 29]) of CP untrained while their non-dominant side showed worse values compared with TD untrained (-10% [-33; 12]). CP trained showed lower central activation ratios in both legs, with the dominant leg (-14% [-34; 6]) showing worse values than their non-dominant one (-4% [-28; 21]). All data can be found in Table 3; Fig. 2. Data from linear regressions can be found in *Supplementary Material*.

**Table 3:** Voluntary activation and central activation ratios for all study groups. Data for the dominant (non-affected) and non-dominant (affected) leg are presented as mean with SD

	CP Trained (*n* = 4)		CP Untrained (*n* = 4)		TD Trained (*n* = 8)		TD Untrained (*n* = 8)	
Voluntary activation [%]	Dominant (*n* = 4)	Non-dominant (*n* = 4)	Dominant (*n* = 4)	Non-dominant (*n* = 4)	Dominant (*n* = 8)	Non-dominant (*n* = 8)	Dominant (*n* = 8)	Non-dominant (*n* = 8)
	75.4 (5.0)	69.5 (10.2)	73.7 (3.3)	76.0 (3.5)	87.6 (7.9)	85.7 (7.0)	86.0 (5.4)	79.8 (11.3)
Central activation ratio [%]	Dominant (*n* = 5)	Non-dominant (*n* = 5)	Dominant (*n* = 4)	Non-dominant (*n* = 4)	Dominant (*n* = 9)	Non-dominant (*n* = 9)	Dominant (*n* = 9)	Non-dominant (*n* = 9)
	71.1 (21.1)	79.0 (13.4)	73.6 (16.4)	78.4 (19.6)	92.1 (5.4)	87.8 (13.2)	82.2 (11.1)	79.8 (10.9)

**Fig. 3:**
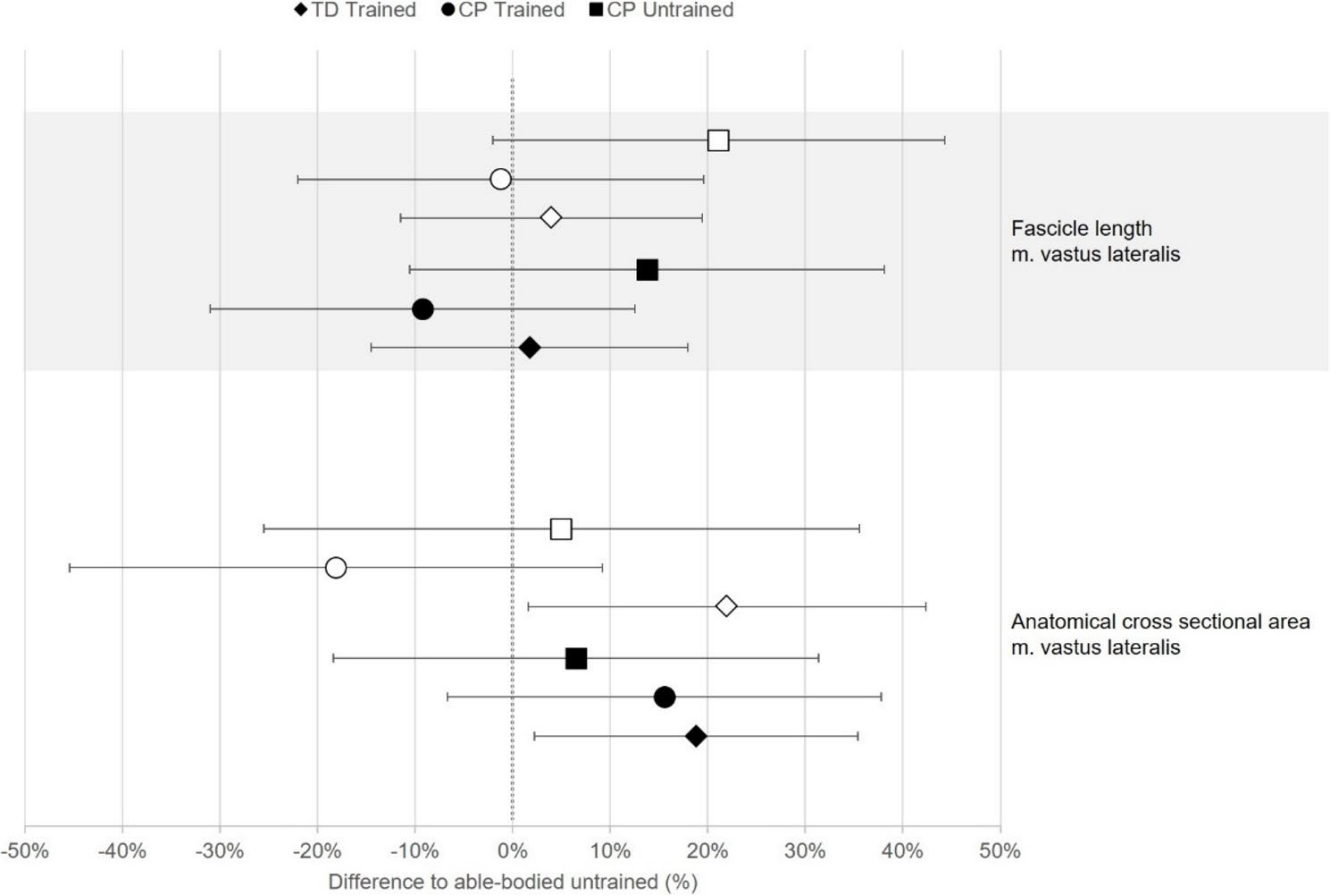
Between-group differences voluntary activation and central activation ratio from linear regression model. Parameters shown as differences in percent to typically-developed untrained group. Dominant leg = black; Non-dominant leg = white; TD = typically-developed; CP = cerebral palsy.

### Muscle geometry

**Fig. 4:**
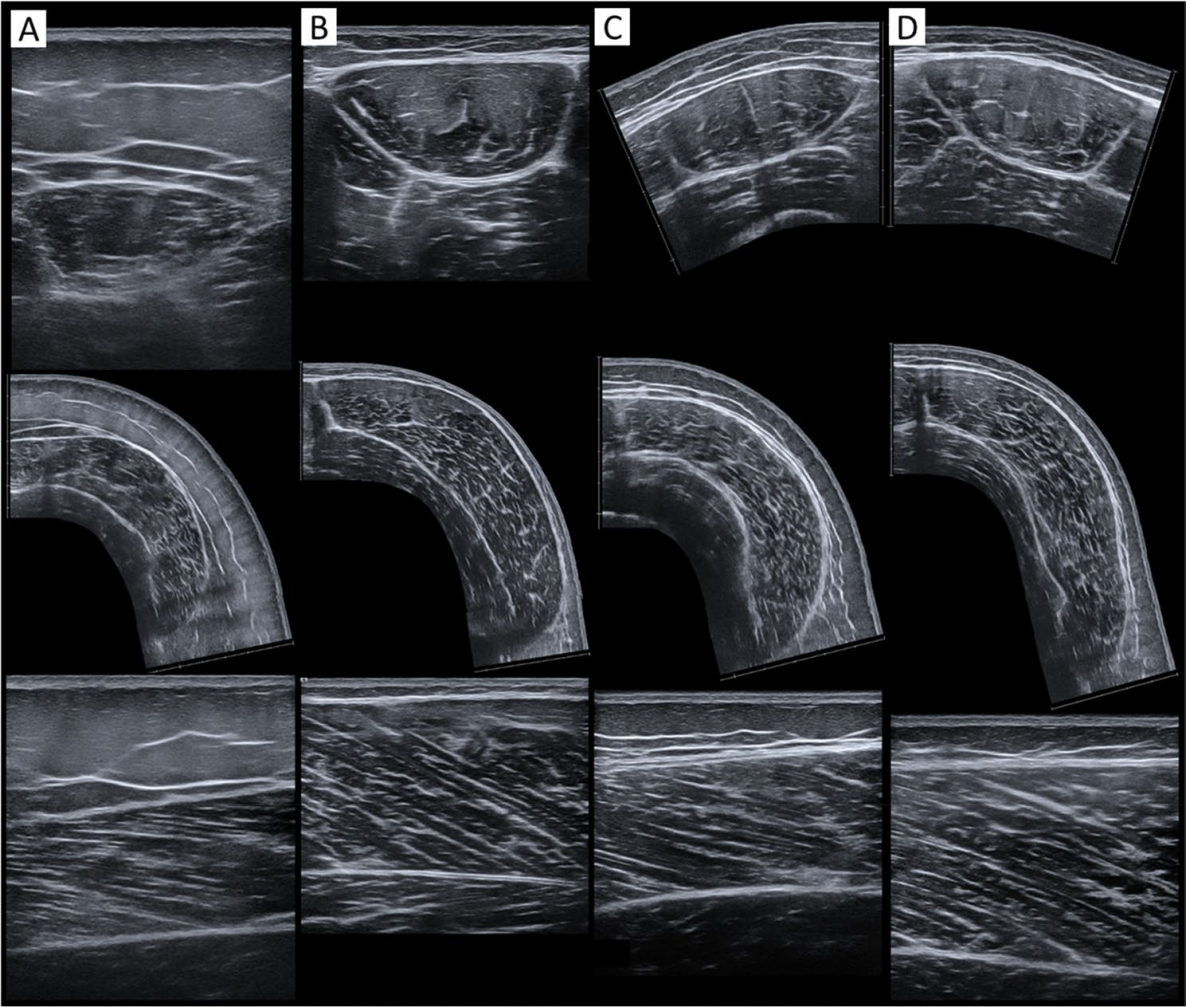
Panoramic (top and middle row) and static (bottom row) Ultrasonography images of the m. rectus femoris (top row) and m. Vastus lateralis (middle row) anatomical cross-sectional area as well as m. vastus lateralis architecture (bottom row) of cerebral palsy untrained (**A**), cerebral palsy trained (**B**), healthy untrained (**C**) and healthy trained (**D**) females. The images were taken at 50% of femur length in the dominant (left) leg of the participants

CP trained showed higher ACSA than TD untrained in the dominant leg of the m. vastus lateralis (+ 16% [-7; 38]), while the non-dominant side showed lower values (-18% [-45; 9]). Side-differences in ACSA of m. vastus lateralis were less pronounced in both TD trained (+ 19 vs. + 22%) as well as CP untrained (+ 6 vs. + 5%) (Fig. 3). CP trained showed higher PA (dominant: +26% [2; 50]; non-dominant: +9% [-12; 30]) and MT (dominant: +9% [-19; 38]; non-dominant: +13% [-12; 38]) than TD untrained. CP untrained also showed higher MT (dominant: +22% [-10; 54]; non-dominant: +20% [-8; 48]) than TD untrained while PA was lower in their non-dominant (-20% [-44; 3]) but not dominant leg (+ 6% [-21; 33]). Representative ultrasonography images showing ACSA and MT are presented in Figure 4. Data can be found in Table 4; Fig. 3. Linear regression outcomes can be found in *Supplementary Material*. Fig. 4.

**Table 4:** Data from muscle ultrasound imaging for all groups. Data for the dominant (non-affected) and non-dominant (affected) leg are presented as mean with SD

	CP Trained (*n* = 5)		CP Untrained (*n* = 4)		Typically-developed Trained (*n* = 9)		Typically-developed Untrained (*n* = 9)	
m. vastus lateralis	Dominant	Non-dominant	Dominant	Non-dominant	Dominant	Non-dominant	Dominant	Non-dominant
Anatomical cross-sectional area [cm^2^]	31.3 (7.6)	22.9 (4.8)	20.4 (4.6)	20.0 (4.4)	28.2 (7.4)	28.6 (8.5)	23.7 (5.8)	23.4 (5.6)
Pennation angle [°]	23.8 (3.8)	21.9 (3.0)	18.3 (3.3)	15.8 (3.4)	22.8 (2.6)	20.5 (2.5)	18.3 (3.3)	20.0 (2.9)
Fascicle length [mm]	77.5 (5.8)	79.7 (1.4)	80.6 (23.7)	84.4 (22.4)	79.4 (12.1)	78.2 (10.1)	78.1 (12.7)	75.2 (11.3)
Muscle thickness [mm]	30.3 (8.0)	31.0 (6.7)	24.5 (5.5)	24.3 (4.4)	27.6 (4.9)	25.8 (4.5)	24.2 (6.3)	24.2 (6.7)
m. rectus femoris	Dominant	Non-dominant	Dominant	Non-dominant	Dominant	Non-dominant	Dominant	Non-dominant
Anatomical cross-sectional area [cm^2^]	8.7 (2.0)	6.2 (0.6)	5.8 (1.5)	5.8 (1.5)	7.1 (1.6)	6.9 (2.1)	8.0 (2.0)	7.8 (2.4)

## Discussion

The aim of the present study was to examine differences in strength, voluntary activation and muscle geometry in individuals with CP compared to TD peers in dependence of training status. Our findings show that regular resistance and endurance training exposure is associated with a smaller gap in muscular strength and muscle ACSA in the dominant (i.e. non-affected) leg of individuals with CP but not with reduced neuromuscular deficits.

Strength deficits in individuals with CP have been reported to vary greatly between studies, with the differences lying in the muscles examined, the methods of assessment, the control groups involved and the form of CP as well as the functionality of subjects. A study examining CP populations of the same GMFCS level as our study population measured deficits ranging between 32 and 44% in knee extension and 33–48% in knee flexion, compared with TD peers [31]. A further study examining children with diplegic CP report similar values, with force reductions of 47% in knee extension and reductions of 53% in knee flexion [32]. Our data comparing CP untrained with TD peers is compatible with these previous findings, with relative strength differences ranging between − 25% and − 35%, independently of dominant or non-dominant leg. Largest deficits in the CP untrained group were found for rate of force development, implying that not only do they reach lower absolute strength values, but they also have reduced explosive strength. Our findings in the CP trained group, however, differ notably from the literature and from the CP untrained group. There is a clear discrepancy between their dominant and non-dominant leg, with strength relative to body mass showing 13% lower values in the dominant (i.e. non-affected) leg while the non-dominant (i.e. affected) leg showed a 32% reduced force. As CP untrained did not show such side-differences between affected (-34%) and non-affected (-32%) limbs, we can assume that the increased strength in the dominant leg of the CP trained group is due to their training stimulus. While we assume that this is likely, our data is not able to provide evidence for this due to the cross-sectional design of the study. The observed disparity in strength between the affected and non-affected limbs in the trained CP group, but not in the untrained CP group, suggests that regular training can enhance limb strength. However, this improvement appears to predominantly benefit the non-affected limb in individuals with CP, while the affected limb does not experience comparable gains from long-term training. Our cross-sectional evidence therefore aligns with previous intervention studies in children with spastic CP, showing that strength training regimens led to increased overall strength while simultaneously exacerbating inter-limb strength asymmetries [33].

The side-specific adaptations found for maximum strength are reflected in muscle geometry. Our data shows that ACSA greatly differed between dominant and non-dominant leg, but only in the CP trained group. CP trained showed 19% higher ACSA of the m. vastus lateralis while the non-dominant leg showed a reduced muscle ACSA (-18%) compared with TD untrained peers. This anatomical alteration was not evident in CP untrained, which showed similar ACSA of the m. vastus lateralis in their dominant (+ 6%) and non-dominant (+ 5%) leg. The discrepancy between the trained and untrained group shows that training mainly affected muscle growth in the non-affected leg what may be explained by reduced mechanical loads applied to the affected limb during training [34].

Our findings do not fully corroborate the available studies on muscle mass in CP, which show that ACSA can be as much as 48% reduced in individuals with CP compared to TD individuals depending on the examined muscle [15, 35]. However, the smaller differences observed in our study may be explained by the inclusion of individuals with GMFCS levels I and II, which represent mild(er) forms of CP. Alterations in muscle architecture and size have been shown in the m. rectus femoris and m. vastus lateralis in children with CP, potentially playing a major role in decreased capacity for force generation and locomotion, while a systematic review was able to present general consistency indicating muscle volume, CSA, thickness and belly length to be reduced in individuals with spastic CP [14, 15]. This discrepancy in findings can additionally be attributed to our statistical approach, which analyzed matched individuals and did not calculate percentage from absolute group values.

An important finding in previous studies is that architectural differences vary strongly between individuals and especially between functionality in individuals with CP [35]. The level of muscle volume reduction is linked to reduced function and, correspondingly, severity of motor impairment [36, 37]. Handsfield et al. showed that lower limb muscles are not uniformly reduced in volume, implying that the neurological condition does not affect all muscles equally, which further strengthens the notion that treatment should be individually tailored to each affected individual [38]. In individuals with CP, fascicle length has been reported to vary from no difference to reductions of up to 25%, and in some cases greater than 40%. Interestingly, our data shows that the CP trained group tend to have shorter fascicles in their dominant leg (-9%) while the untrained CP group showed up to 21% longer length than TD untrained. Since CP untrained have both higher FL and lower ACSA in both legs, one might assume that muscle volume is also reduced, therefore leading to reduced force production and strength.

The trajectory of typically developing muscle size and strength steadily increases before peaking in the early to mid-20s [39]. Regular exercise training should promote development of muscular function during this period to maintain health above the functional strength threshold for as long as possible and to combat aging and sarcopenia. Individuals with CP report significantly higher sedentary behavior than TD counterparts and, most importantly, entertain fewer opportunities to mechanically load their muscles [40, 41]. Correspondingly, muscle volume trajectories in individuals with CP are characterized by lower peak values, earlier peak occurrence, and a closer proximity to the functional threshold across the lifespan [38]. Our reported geometrical adaptations to regular mechanical load should be regarded as evidence that regular strength training can increase not only muscle ACSA and strength, but more importantly can create a greater distance to the functional threshold, providing valuable reserves later in life.

In this cross-sectional study, we observed decreased strength differences and increased muscle ACSA in the dominant leg in CP trained. In contrast to these data, neuromuscular activation was still impaired when compared to TD untrained. The evidence concerning VA in CP remains scarce with contrasting evidence. For example, O’Brien et al. showed for the plantarflexors that VA was not predictive of strength in individuals with CP with GMFCS level I-III [12]. Based on their data, the authors therefore concluded that reduced muscle size contributes more to weakness than voluntary activation level. However, other authors observed more pronounced impairments in the level of voluntary activation and therefore argued that this impairment is a primary contributor for diminished strength [9]. The authors further argued that children with CP who have large deficits in voluntary muscle activation may not be able to generate sufficient forces during voluntary contractions to induce muscle hypertrophy. This assumption is partly supported by our data, because participants in the CP trained group showed impairments for muscle mass and muscle strength on the non-dominant side while no or reduced impairments were observed for those outcomes in the dominant leg.

We cannot provide a definite explanation for the absence of neural adaptations in the CP trained group. One possible reason is the neurological damage itself, since individuals with CP have increased antagonist coactivation and/or are (often) unable to recruit type II muscle fibers [11]. However, a second possible reason might be the training. It has been shown in healthy adults that high rather than low loads during resistance training are more effective in producing neural changes in the short- but also long-term [34, 42, 43]. With respect to the voluntary activation level, it has been shown that six weeks of high-load resistance training results in an increased level of voluntary activation while low-load training did not induce neural adaptations [44]. Thus, it may be assumed that our training regimen with a focus on low/moderate loads with high repetitions was not sufficient to induce the level of voluntary activation. Further studies applying near-maximum loads are necessary to discern whether high-load resistance training is able to achieve neural adaptations in CP populations.

### Conclusion and practical relevance

Our data confirms the current literature suggesting that weakness in CP arises from neuronal damage and muscular alterations, as evidenced by discrepancies between the affected and non-affected leg in our CP trained group and when comparing individuals with CP to healthy controls. Long-term training exposure potentially closes the strength gap in knee extension strength of the dominant leg in individuals with CP, but not of the non-dominant (affected) leg. Similarly, muscle volume of the dominant leg increased with training while the non-dominant leg did not show signs of hypertrophy. Neural drive does not seem to be affected through long-term training. Thus, the participation of individuals with CP in long-term high-intensity training mitigates primarily muscular impairments in the dominant limb. Future studies should investigate if machine-based and one-legged training is more effective in eliciting adaptations in the non-dominant (and more severely affected) limb.

Exercise recommendations for CP populations aim at increasing muscle mass and strength to increase independence and movement in daily life while simultaneously reducing physical limitations and secondary health issues [18]. Our data shows how a long-term implementation of the exercise guidelines can increase strength and ACSA in the non-affected legs of adolescents with CP, but does not alter voluntary activation. Despite attending the training sessions, the CP trained population still show low levels of moderate-to-vigorous activity levels (194 min/week) compared with the TD trained group (503 min/week). This implies that the transfer effect into daily living is not given by following exercise recommendations. Whether the absence of transfer-effect is due to environmental or personal and motivational factors, remains unknown. While the improvement of strength in the non-affected limb does ameliorate the risk factor of weakness and provides an important reserve for the functional threshold, it does not seem sufficient to increase habitual activity. Future challenges for training interventions or training programs for individuals with CP remain in finding training methods which can elicit improvements in the affected limbs while also increasing habitual activity levels.

### Limitations

The rigorous inclusion criteria defined in the feasibility study limited the number of patients who participated in the training intervention and who continued the training regimen until the present follow-up. Despite the relatively small sample size, the investigatory nature of this study remains highly relevant, especially considering the sex- and age-matched groups as well as being the first study examining a population of trained individuals with CP. Due to the inclusion of patients with mild(er) impairments (*n* = 8 GMFCS level I; *n* = 1 GMFCS level II), the results demonstrated in this study are not broadly applicable to more severe types of CP. This is especially true since patients with GMFCS level III to level V typically depend on hand-held mobility devices or may even rely on a wheelchair for mobility. Thus, high-intensity training may not be possible in patients with more severe forms of CP.

Ultrasonography allowed assessment of ACSA in one location, and we were not able to measure muscle volume, regional differences or muscle contractility which could provide deeper insight into muscle function. The present data should be interpreted with caution due to the level of uncertainty related to large confidence intervals. Another limitation is that we were not able to control for all potential confounders when recruiting the typically developing participants. Factors such as hormonal levels, alcohol and substance use, socioeconomic background, dietary intake (i.e. protein and overall caloric intake), or medication may influence the study outcomes, yet were not accounted for in our analysis.

## Supplementary Information

Supplementary Material 1

## Data Availability

The datasets used and/or analysed during the current study are available from the corresponding author on reasonable request.
