# Supplementary material for: The relationship between exposure to long-term training, neuromuscular function and muscular structure in adolescents with cerebral palsy and typically-developed peers: a cross-sectional follow-up analysis
Source: BMC Musculoskelet Disord. 2026 May 1;27:531. doi: 10.1186/s12891-026-09829-3 (PMC13281589; doi:10.1186/s12891-026-09829-3)
Supplement: Supplementary file 1 — Supplementary Material 1 [file 12891_2026_9829_MOESM1_ESM.pdf]

Supplementary Material: Outcomes from linear regression models, comparing sex- and age-matched differences of CP trained, CP untrained and typically-developed trained to typically-developed untrained. Between-differences are given as standardized percentage from typically-developed untrained (reference group) with 95% confidence intervals (95% CI).

**Isometric knee extension 60°**		**Dominant**		**Non Dominant**	
Max. strength	Typically-developed Trained	+7%	[-15;29]	+5%	[-13;22]
	Cerebral Palsy Trained	-18%	[-48;11]	-35%	[-58;-11]
	Cerebral Palsy Untrained	-29%	[-61;4]	-33%	[-59;-7]
Strength relative body mass	Typically-developed Trained	+18%	[-11;46]	+16%	[-8;41]
	Cerebral Palsy Trained	-13%	[-52;25]	-32%	[-65;0]
	Cerebral Palsy Untrained	-32%	[-75;11]	-34%	[-71;3]
Strength relative lean mass	Typically-developed Trained	+1%	[-22;24]	0%	[-19;19]
	Cerebral Palsy Trained	-17%	[-48;14]	-34%	[-59;-8]
	Cerebral Palsy Untrained	-31%	[-65;4]	-33%	[-62;-5]
Rate of force development	Typically-developed Trained	-24%	[-65;18]	-21%	[-47;6]
	Cerebral Palsy Trained	-15%	[-70;41]	-29%	[-65;7]
	Cerebral Palsy Untrained	-73%	[-135;-11]	-74%	[-114;-34]
**Isokinetic knee extension / flexion**		**Dominant**		**Non Dominant**	
Extension relative body mass	Typically-developed Trained	+13%	[-12;43]	+24%	[-7;50]
	Cerebral Palsy Trained	-14%	[-51;24]	-36%	[-75;2]
	Cerebral Palsy Untrained	-35%	[-75;4]	-27%	[-70;16]
Extension relative lean mass	Typically-developed Trained	0%	[-27;25]	+3%	[-23;31]
	Cerebral Palsy Trained	-17%	[-53;18]	-39%	[-75;-3]
	Cerebral Palsy Untrained	-35%	[-75;4]	-26%	[-67;14]
Flexion relative body mass	Typically-developed Trained	+25%	[-8;52]	+25%	[-4;53]
	Cerebral Palsy Trained	-9%	[-49;32]	-25%	[-64;14]
	Cerebral Palsy Untrained	-31%	[-77;14]	-26%	[-69;18]
Flexion relative lean mass	Typically-developed Trained	0%	[-22;29]	+6%	[-17;29]
	Cerebral Palsy Trained	-12%	[-47;23]	-26%	[-57;4]
	Cerebral Palsy Untrained	-28%	[-67;11]	-25%	[-59;10]
**Twitch interpolation**		**Dominant**		**Non Dominant**	
Voluntary activation	Typically-developed Trained	+2%	[-5;9]	+7%	[-7;22]
	Cerebral Palsy Trained	-13%	[-23;-3]	-10%	[-30;11]
	Cerebral Palsy Untrained	-14%	[-23;-4]	-8%	[-29;13]

Central activation ratio	Typically-developed Trained	12%	[-3;27]	10%	[-8;28]
	Cerebral Palsy Trained	-14%	[-34;6]	-4%	[-28;21]
	Cerebral Palsy Untrained	-10%	[-33;12]	1%	[-26;29]
**M. vastus lateralis geometry**		**Dominant**		**Non Dominant**	
Anatomical cross-sectional area	Typically-developed Trained	+19%	[2;35]	+22%	[2;42]
	Cerebral Palsy Trained	+16%	[-7;38]	-18%	[-45;9]
	Cerebral Palsy Untrained	+6%	[-18;31]	+5%	[-26;36]
Pennation angle	Typically-developed Trained	+24%	[6;42]	+2%	[-13;18]
	Cerebral Palsy Trained	+26%	[2;50]	+9%	[-12;30]
	Cerebral Palsy Untrained	+6%	[-21;33]	-20%	[-44;3]
Fascicle length	Typically-developed Trained	+2%	[-14;18]	+4%	[-11;19]
	Cerebral Palsy Trained	-9%	[-31;13]	-1%	[-22;20]
	Cerebral Palsy Untrained	+14%	[-11;38]	+21%	[-2;44]
Muscle thickness	Typically-developed Trained	+14%	[-7;36]	+7%	[-12;25]
	Cerebral Palsy Trained	+9%	[-19;38]	+13%	[-12;38]
	Cerebral Palsy Untrained	+22%	[-10;54]	+20%	[-8;48]
**M. rectus femoris geometry**		**Dominant**		**Non Dominant**	
Anatomical cross-sectional area	Typically-developed Trained	-11%	[-24;3]	-11%	[-28;6]
	Cerebral Palsy Trained	-1%	[-19;18]	-31%	[-54;-8]
	Cerebral Palsy Untrained	-16%	[-36;5]	-11%	[-37;14]
